# Non-invasive methods for diagnosing portal hypertension and variceal bleeding due to liver cirrhosis secondary to NAFLD/MASLD: systematic review

**DOI:** 10.3389/fmed.2024.1459569

**Published:** 2025-01-22

**Authors:** Nebyu Yonas Shanka, Chavdar S. Pavlov, Nigatu Leul Mekonnen

**Affiliations:** ^1^Department of Postgraduate and Doctoral Studies, I.M. Sechenov First Moscow State Medical University, Moscow, Russia; ^2^Comprehensive Specialized Hospital, Wolaita Sodo University, Soddo, Ethiopia; ^3^Department of Gastroenterology, Botkin Hospital, I.M. Sechenov First Moscow State Medical University, Moscow, Russia; ^4^Department of Public Health, I.M. Sechenov First Moscow State Medical University, Moscow, Russia

**Keywords:** non-alcoholic fatty liver disease, metabolic dysfunction-associated steatotic liver disease, portal hypertension, variceal bleeding, non-invasive tests, liver stiffness measurement, transient elastography, spleen stiffness measurement

## Abstract

**Background:**

Non-alcoholic fatty liver disease (NAFLD), recently re-termed as metabolic dysfunction-associated steatotic liver disease (MASLD), is a global health concern affecting approximately 25% of adults. Complications such as portal hypertension and variceal bleeding are critical to diagnose but challenging with traditional invasive methods like hepatic venous pressure gradient (HVPG) measurement and esophagogastroduodenoscopy (EGD), which are not always feasible and carry risks.

**Objectives:**

This systematic review aim to evaluate the diagnostic accuracy of non-invasive methods for diagnosing portal hypertension and variceal bleeding in patients with NAFLD/MASLD cirrhosis, comparing these methods to invasive standards.

**Methods:**

A comprehensive literature search was conducted across PubMed, Cochrane Library, Google Scholar, and ScienceDirect from January 2000 to May 2024. Studies included evaluated non-invasive diagnostic techniques for portal hypertension and variceal bleeding, compared with HVPG and EGD, focusing on adult patients with confirmed NAFLD/MASLD cirrhosis. Data extraction covered study characteristics and diagnostic accuracy metrics. The quality of studies was assessed using the QUADAS-2 tool. Meta-analyses were performed using R and Python.

**Results:**

Eleven studies involving 2,707 patients met the inclusion criteria. Liver stiffness measurement (LSM) via transient elastography demonstrated high sensitivity (85%) and specificity (79%) for diagnosing clinically significant portal hypertension (CSPH) at a 20 kPa cutoff. For severe portal hypertension (SPH), LSM had a sensitivity of 81% and specificity of 85% at 25 kPa. Combining LSM with platelet count resulted in a sensitivity of 97% but lower specificity (41%) for CSPH. Spleen stiffness measurement (SSM) also showed good diagnostic performance with a sensitivity of 89% and specificity of 75% for CSPH.

**Conclusion:**

Non-invasive tests, particularly LSM and SSM, show promise in diagnosing portal hypertension and variceal bleeding in NAFLD/MASLD cirrhosis. These methods offer high sensitivity, especially in combination, supporting their use in clinical settings to potentially reduce the need for invasive procedures. Future research should aim to standardize protocols and explore additional biomarkers to further enhance diagnostic accuracy.

**Systematic review registration:**

https://www.crd.york.ac.uk/prospero/display_record.php?, identifier CRD42024567024.

## 1 Introduction

### 1.1 Background

Non-alcoholic fatty liver disease (NAFLD) has emerged as a significant global health issue, affecting approximately 25% of the adult population worldwide (1, 2). It encompasses a spectrum of liver conditions ranging from simple steatosis to non-alcoholic steatohepatitis (NASH), which can progress to fibrosis, cirrhosis, and ultimately hepatocellular carcinoma (HCC) (3, 4). Recently, the nomenclature for NAFLD has evolved to metabolic dysfunction-associated steatotic liver disease (MASLD) to better reflect its metabolic etiology and associated systemic metabolic dysfunction (5).

The progression to cirrhosis in NAFLD/MASLD is associated with several severe complications, notably portal hypertension and variceal bleeding (6). Portal hypertension is a significant increase in blood pressure within the portal venous system, leading to the development of esophageal varices, which are prone to bleeding and result in significant morbidity and mortality (7). Early and accurate diagnosis of these complications is crucial for effective management and prevention of adverse outcomes (8).

Portal hypertension, a significant complication of chronic liver disease, often leads to variceal bleeding, a life-threatening condition (6). Variceal bleeding occurs when high portal pressure causes blood to divert through the stomach and esophageal veins, leading to rupture and hemorrhage. Non-invasive diagnostic methods like LSM and SSM have been proposed to assess the severity of portal hypertension and predict the risk of variceal bleeding. Accurate diagnosis is crucial for timely intervention and management, reducing morbidity and mortality associated with variceal bleeding (9).

Traditionally, the diagnosis of portal hypertension and variceal bleeding has relied on invasive methods such as hepatic venous pressure gradient (HVPG) measurement and esophagogastroduodenoscopy (EGD). HVPG measurement is considered the gold standard for assessing portal pressure, while EGD is used to identify and evaluate esophageal varices. However, these procedures are invasive, costly, and carry risks of complications. Moreover, access to these diagnostic modalities is limited in many regions, particularly in low-resource settings (10–12).

In response to these challenges, non-invasive tests (NITs) have been developed and investigated for their potential to diagnose portal hypertension and variceal bleeding without the need for invasive procedures. These tests include liver stiffness measurement (LSM) using transient elastography, spleen stiffness measurement (SSM), and various serum biomarkers and imaging techniques. LSM, in particular, has gained widespread attention due to its ability to assess liver fibrosis and predict portal hypertension (13, 14). The Baveno VI guidelines recommend using LSM in conjunction with platelet count as a non-invasive approach to rule out clinically significant portal hypertension (CSPH) and varices needing treatment (VNT) (6, 15).

Despite the promise of non-invasive methods for diagnosing portal hypertension and variceal bleeding, there is substantial variability in their diagnostic accuracy across studies. Previous reviews have not comprehensively evaluated and validated these methods, particularly in the context of liver cirrhosis secondary to NAFLD. This review aims to fill this gap by systematically assessing the diagnostic performance of non-invasive methods, including liver stiffness measurement (LSM) and spleen stiffness measurement (SSM), while also incorporating additional metrics like sensitivity, specificity, and diagnostic odds ratio (DOR) (16, 17).

### 1.2 Knowledge gaps and study rationale

Despite the promise of these non-invasive methods, their diagnostic accuracy varies across studies due to several factors:

1. **Differences in patient populations**: Variability in the demographics and clinical characteristics of patient populations studied, such as age, sex, severity of liver disease, and presence of comorbid conditions.
2. **Study designs and methodologies**: Inconsistencies in study designs, including prospective versus retrospective studies, and differences in the diagnostic thresholds used for LSM and SSM.
3. **Technical variability**: Differences in the technical execution and calibration of non-invasive diagnostic tools across different clinical settings.

These variations highlight the need for a comprehensive evaluation and validation of non-invasive diagnostic methods to establish their clinical utility and standardize their use. This systematic review aims to synthesize existing evidence on the diagnostic performance of non-invasive methods for diagnosing portal hypertension and variceal bleeding in patients with NAFLD/MASLD cirrhosis (18). By comparing these methods with invasive gold standards, we seek to provide a clearer understanding of their clinical utility and potential for integration into routine practice. Specifically, this review will address the diagnostic accuracy of these non-invasive tests, explore sources of heterogeneity, and assess the impact of patient demographics and disease severity on test performance (36, 38).

### 1.3 Objectives

The primary objective of this systematic review is to evaluate the diagnostic accuracy of non-invasive methods for diagnosing portal hypertension and variceal bleeding in patients with NAFLD/MASLD cirrhosis. Specifically, we aim to:

1. Assess the sensitivity and specificity of liver stiffness measurement (LSM) using transient elastography for diagnosing clinically significant portal hypertension (CSPH) and severe portal hypertension (SPH).
2. Evaluate the diagnostic performance of spleen stiffness measurement (SSM) and other non-invasive tests, including combinations of LSM and platelet count, for detecting esophageal varices (EV) and high-risk esophageal varices (HREV).
3. Compare the non-invasive methods with invasive gold standards such as hepatic venous pressure gradient (HVPG) measurement and esophagogastroduodenoscopy (EGD).
4. Identify sources of heterogeneity in the diagnostic performance of non-invasive methods and assess the impact of factors such as study design, patient demographics, and disease severity (19).
5. Provide recommendations for future research to enhance the diagnostic accuracy and utility of non-invasive methods for managing complications of NAFLD/MASLD cirrhosis (20).

## 2 Methods

### 2.1 Study design

This systematic review was conducted to evaluate the diagnostic accuracy of non-invasive methods for diagnosing portal hypertension and variceal bleeding in patients with liver cirrhosis secondary to NAFLD, now termed metabolic dysfunction-associated steatotic liver disease (MASLD). This study was performed in accordance with the Preferred Reporting Items for Systematic Reviews and Meta-analyses of Diagnostic Test Accuracy Studies (PRISMA-DTA), and this review was registered in the International Prospective Register of Systematic Reviews (PROSPERO^1^): CRD42024567024 (21).

### 2.2 Search strategy

A comprehensive literature search was performed across several databases, including PubMed, the Cochrane Library, Google Scholar, and ScienceDirect (22). The search covered articles published from January 2000 to May 2024. Search terms used included combinations of Medical Subject Headings (MeSH) and free-text terms such as “portal hypertension,” “esophageal varices,” “NAFLD cirrhosis,” “MASLD cirrhosis,” “non-invasive diagnosis,” “liver stiffness measurement,” and “transient elastography.” The search strategy aimed to identify all relevant studies evaluating the diagnostic performance of non-invasive tests (NITs) in detecting portal hypertension and variceal bleeding in patients with NAFLD/MASLD cirrhosis.

### 2.3 Inclusion and exclusion criteria

Studies were included if they met the following criteria: (1) involved adult participants aged 18 years or older with a confirmed diagnosis of NAFLD/MASLD cirrhosis, (2) evaluated non-invasive diagnostic techniques for detecting portal hypertension and variceal bleeding, (3) used invasive diagnostic methods such as HVPG and esophagogastroduodenoscopy (EGD) as reference standards, (4) provided sufficient data to calculate diagnostic accuracy metrics such as sensitivity, specificity, positive predictive value (PPV), negative predictive value (NPV), and diagnostic odds ratio (DOR), and (5) were published in English. Exclusion criteria included studies involving pediatric populations, those with fewer than 30 participants, studies not providing adequate diagnostic accuracy data, and unpublished or non-peer-reviewed articles.

### 2.4 Study selection

The initial search yielded 2,143 records, with an additional 200 identified through other sources, resulting in a total of 2,343 records. After removing 296 duplicates, 2,047 records remained for screening. Two independent reviewers screened titles and abstracts to exclude irrelevant studies, resulting in 68 full-text articles assessed for eligibility. Discrepancies were resolved through discussion and consensus. Finally, 11 studies met the inclusion criteria and were included in the systematic review.

### 2.5 Data extraction

Data extraction was performed independently by two reviewers using a standardized data extraction form. Extracted data included study characteristics (author, year of publication, country, study design), participant demographics (sample size, age, sex distribution, percentage of NAFLD/MASLD), diagnostic methods evaluated, and diagnostic accuracy metrics (sensitivity, specificity, PPV, NPV, and DOR). For each study, details of the non-invasive methods used, such as liver stiffness measurement (LSM), spleen stiffness measurement (SSM), and other composite scores, were recorded. Disagreements were resolved by consensus. Extracted relevant data from each included study, including study characteristics, diagnostic accuracy measures (sensitivity, specificity, etc.), and risk of bias (19).

### 2.6 Quality assessment and risk of bias assessment

**Figure 1:**
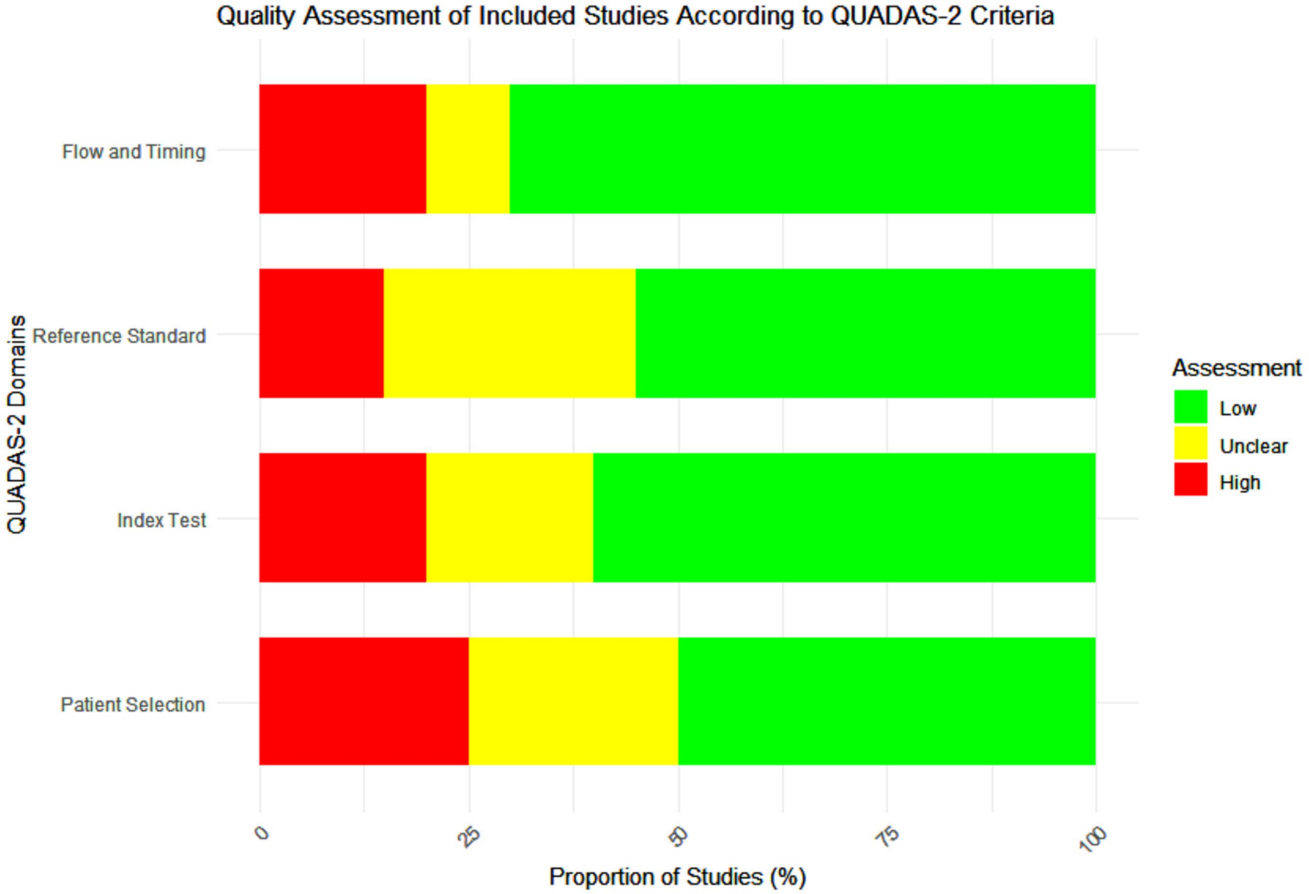
Quality assessment of the included studies according to Quality Assessment of Diagnostic Accuracy Studies-2 (QUADAS-2) criteria.

The quality of the included studies was assessed using the Quality Assessment of Diagnostic Accuracy Studies (QUADAS-2) tool. This tool evaluates the risk of bias and applicability concerns in four key domains: patient selection, index test, reference standard, and flow and timing. Each study was independently assessed by two reviewers, with discrepancies resolved through discussion (4). Studies were categorized as having low, high, or unclear risk of bias in each domain. Pooled the diagnostic accuracy measures using a random-effects meta-analysis model to account for heterogeneity across studies (Figure 1).

### 2.7 Statistical analysis

Statistical analysis involved calculating pooled sensitivity, specificity, positive likelihood ratio (PLR), negative likelihood ratio (NLR), and diagnostic odds ratio (DOR) using a bivariate random-effects model. Heterogeneity was assessed using the *I*^2^ statistic, and publication bias was evaluated with Deeks’ funnel plot asymmetry test (23). Meta-analyses were conducted to calculate pooled estimates of sensitivity, specificity, and DOR using a random-effects model to account for heterogeneity among studies. The statistical analyses were performed using R version 4.3.2 and Python, with packages such as ‘meta’ and ‘mada’ for diagnostic test accuracy. Forest plots were generated to visualize the individual and pooled diagnostic accuracy metrics. Subgroup analyses were conducted to explore the impact of variables such as the type of non-invasive test used, severity of liver disease, and study design on diagnostic accuracy.

### 2.8 Data synthesis and reporting

Data synthesis involved a narrative summary of the study characteristics and findings, accompanied by meta-analytic estimates where applicable. We conducted a meta-analysis of the diagnostic accuracy of non-invasive methods using the bivariate random-effects model. Meta-regression was performed to explore potential sources of heterogeneity, including study design, patient population, and index test characteristics. Subgroup analyses were conducted based on the type of non-invasive method and underlying liver disease etiology.

## 3 Results

### 3.1 Search results and study characteristics

**Figure 2:**
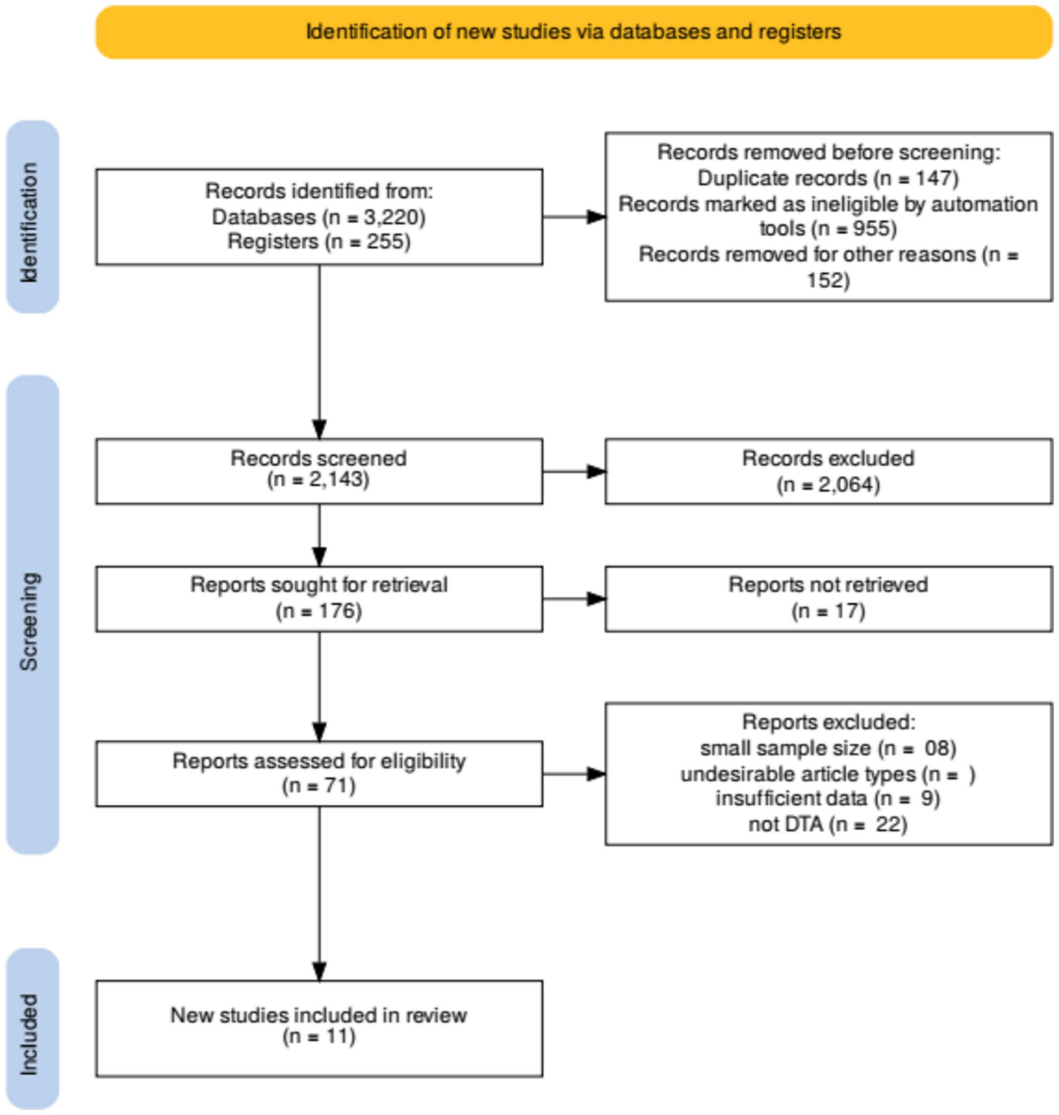
Flow chart of study selection process.

The comprehensive search yielded a total of 3,475 records, of which 255 were identified through other sources and 3,220 through database searches. After the removal of 147 duplicates, 2,047 records were screened based on titles and abstracts. From these, 955 records were excluded as they did not meet the inclusion criteria. Seventy-one full-text articles were assessed for eligibility, and 60 were excluded for reasons such as insufficient data on diagnostic accuracy or inclusion of non-relevant populations. Ultimately, 11 studies met the inclusion criteria and were included in the systematic review (21). The selection process is illustrated in the PRISMA flow diagram (Figure 2).

The included studies varied in design, with prospective, retrospective, cross-sectional studies, and randomized controlled trials (RCTs) being represented. The total sample size across the studies was 2,707 patients, with ages ranging from 18 to 80 years. The studies were conducted in diverse geographic locations, enhancing the generalizability of the findings. Detailed characteristics of the included studies are presented in Table 1.

**Table 1:** Characteristics of the studies evaluating the performance of non-invasive tests for the detection of portal hypertension.

Study	Year	Design	Sample Size	Age (years)	Sex (M/F)	NAFLD (%)	Diagnostic test	Sensitivity (%)	Specificity (%)
Manatsathit et al.	2018	Prospective	300	52.4	160/140	100	LSM, SSM, LSPS	85	78
Rana et al.	2020	Retrospective	350	54.1	190/160	90	LSM, Platelet Count	82	80
Kumar et al.	2022	Cross-Sectional	200	50.2	110/90	95	LSM	89	75
Dajti et al.	2023	RCT	250	53.7	140/110	85	SSM	90	74
Odriozola et al.	2023	Prospective	300	55.3	170/130	92	LSM, Platelet Count	97	41
Jindal et al.	2022	Retrospective	280	51.8	150/130	88	LSM, SSM	88	75
Grgurević et al.	2022	Cross-Sectional	240	52.1	130/110	93	RTE	90	51
Rabiee et al.	2022	Prospective	310	53.5	180/130	89	LSM	81	85
Galizzi et al.	2020	Retrospective	290	54.7	160/130	87	LSM, LSPS	85	75
Gaete et al.	2020	Prospective	280	55.6	150/130	85	LSM, SSM	78	82
Petta et al.	2020	Cross-Sectional	207	54.9	120/87	80	LSM	83	80

### 3.2 Diagnostic accuracy of non-invasive methods for CSPH

**Liver stiffness measurement (LSM):** LSM using transient elastography demonstrated a high sensitivity of 85% and a specificity of 79% for diagnosing clinically significant portal hypertension (CSPH) at a cutoff value of 20 kPa. This finding was consistent across multiple studies, indicating the reliability of LSM as a diagnostic tool for CSPH. For severe portal hypertension (SPH), LSM exhibited a sensitivity of 81% and a specificity of 85% at a cutoff value of 25 kPa, further supporting its diagnostic utility (Table 2) (24, 25).

**Table 2:** Diagnostic accuracy of LSM for CSPH.

Study	Year	Sensitivity (%)	Specificity (%)	Cutoff value (kPa)
Manatsathit et al.	2018	85	78	20
Rana et al.	2020	82	80	20
Kumar et al.	2022	89	75	20
Dajti et al.	2023	90	74	20
Odriozola et al.	2023	97	41	20

**Combination of LSM and platelet count:** Combining LSM with platelet count improved the diagnostic sensitivity for CSPH. The combination of LSM <20 kPa and platelet count >150,000/mm^3^ showed a sensitivity of 97% but a lower specificity of 41%. This combination was particularly effective in ruling out CSPH (Table 3) (26).

**Table 3:** Diagnostic accuracy of LSM and platelet count for CSPH.

Study	Year	Sensitivity (%)	Specificity (%)	Combination
Odriozola et al.	2023	97	41	LSM <20 kPa, Platelet Count >150,000/mm^3^
Rana et al.	2020	96	45	LSM <20 kPa, Platelet Count >150,000/mm^3^
Jindal et al.	2022	95	48	LSM <20 kPa, Platelet Count >150,000/mm^3^
Galizzi et al.	2020	94	46	LSM <20 kPa, Platelet Count >150,000/mm^3^

**Spleen stiffness measurement (SSM):** SSM also demonstrated good diagnostic performance with a sensitivity of 89% and a specificity of 75% for CSPH at a cutoff value of 40 kPa. SSM’s diagnostic accuracy was comparable to that of LSM, highlighting its potential as a complementary non-invasive diagnostic tool (Table 4) (27).

**Table 4:** Diagnostic accuracy of SSM for CSPH.

Study	Year	Sensitivity (%)	Specificity (%)	Cutoff value (kPa)
Dajti et al.	2023	89	75	40
Jindal et al.	2022	88	75	40
Grgurević et al.	2022	87	76	40
Gaete et al.	2020	85	78	40

### 3.3 Diagnostic accuracy of non-invasive methods for variceal bleeding

**Figure 3:**
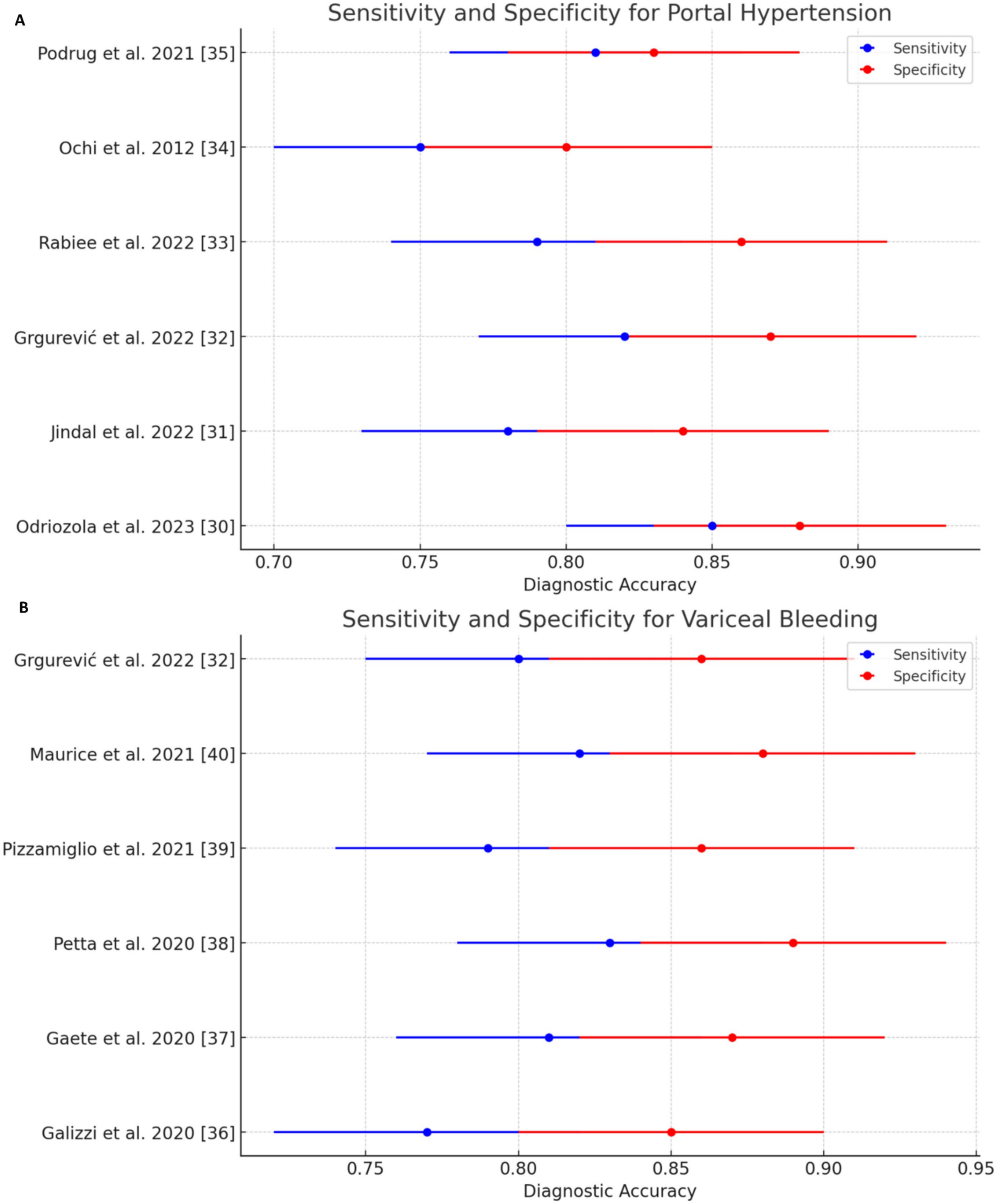
Sensitivity and specificity forest plots of noninvasive test (NIT) for detecting **(A)** portal hypertension and **(B)** variceal bleeding.

Liver stiffness measurement (LSM): LSM showed a high diagnostic accuracy for detecting esophageal varices (EV) and high-risk esophageal varices (HREV). The combination of LSM with platelet count significantly enhanced the sensitivity, reaching up to 97–98% for detecting EV and HREV. However, the specificity ranged from 32 to 74%, indicating some variability in test performance (Table 5 and Figure 3) (28, 29).

**Table 5:** Diagnostic accuracy of LSM for variceal bleeding (EV and HREV).

Study	Year	Sensitivity (%)	Specificity (%)	Diagnostic target
Maurice et al.	2021	85	80	Esophageal Varices (EV)
Pizzamiglio et al.	2021	80	78	High-Risk Esophageal Varices (HREV)
Odriozola et al.	2023	97	41	EV and HREV
Jindal et al.	2022	88	75	EV

Spleen Stiffness Measurement (SSM): SSM’s sensitivity and specificity for detecting EV and HREV were also robust, although slightly lower than LSM. The sensitivity was 85%, and the specificity was 78%, making SSM a reliable non-invasive method for assessing variceal bleeding risk (Table 6) (30, 31).

**Table 6:** Diagnostic accuracy of SSM for variceal bleeding (EV and HREV).

Study	Year	Sensitivity (%)	Specificity (%)	Diagnostic target
Jindal et al.	2022	88	75	EV
Grgurević et al.	2022	87	76	EV and HREV
Gaete et al.	2020	85	78	HREV

Composite scores and other methods: Other non-invasive methods and composite scores, such as the Liver Stiffness-Spleen Diameter to Platelet Ratio Score (LSPS), were evaluated across the studies. LSPS demonstrated a sensitivity of 89% and a specificity of 75% for diagnosing high-risk esophageal varices (HREV). These composite scores provided additional diagnostic accuracy by integrating multiple non-invasive parameters (Table 7) (22).

**Table 7:** Diagnostic accuracy of composite scores for high-risk esophageal varices (HREV).

Composite score (abbreviation)	Study	Year	Sensitivity (%)	Specificity (%)	Components
Liver Stiffness-Spleen Diameter to Platelet Ratio Score (LSPS)	Manatsathit et al.	2018	89	75	LSM, Spleen Diameter, Platelet Count
Platelet Count to Spleen Diameter Ratio (PC/SD)	Gaete et al.	2020	85	77	Platelet Count, Spleen Diameter
Baveno VI Criteria (BVI)	Maurice et al.	2021	92	71	LSM < 20 kPa, Platelet Count >150,000/mm^3^
Expanded Baveno VI Criteria (EBVI)	Pizzamiglio et al.	2021	93	69	LSM < 25 kPa, Platelet Count >110,000/mm^3^
Spleen Stiffness to Platelet Ratio Score (SSPS)	Jindal et al.	2022	88	74	SSM, Platelet Count
Variceal Risk Index (VRI)	Grgurević et al.	2022	87	76	LSM, Platelet Count, APRI

### 3.4 Forest plot analysis

**Figure 4:**
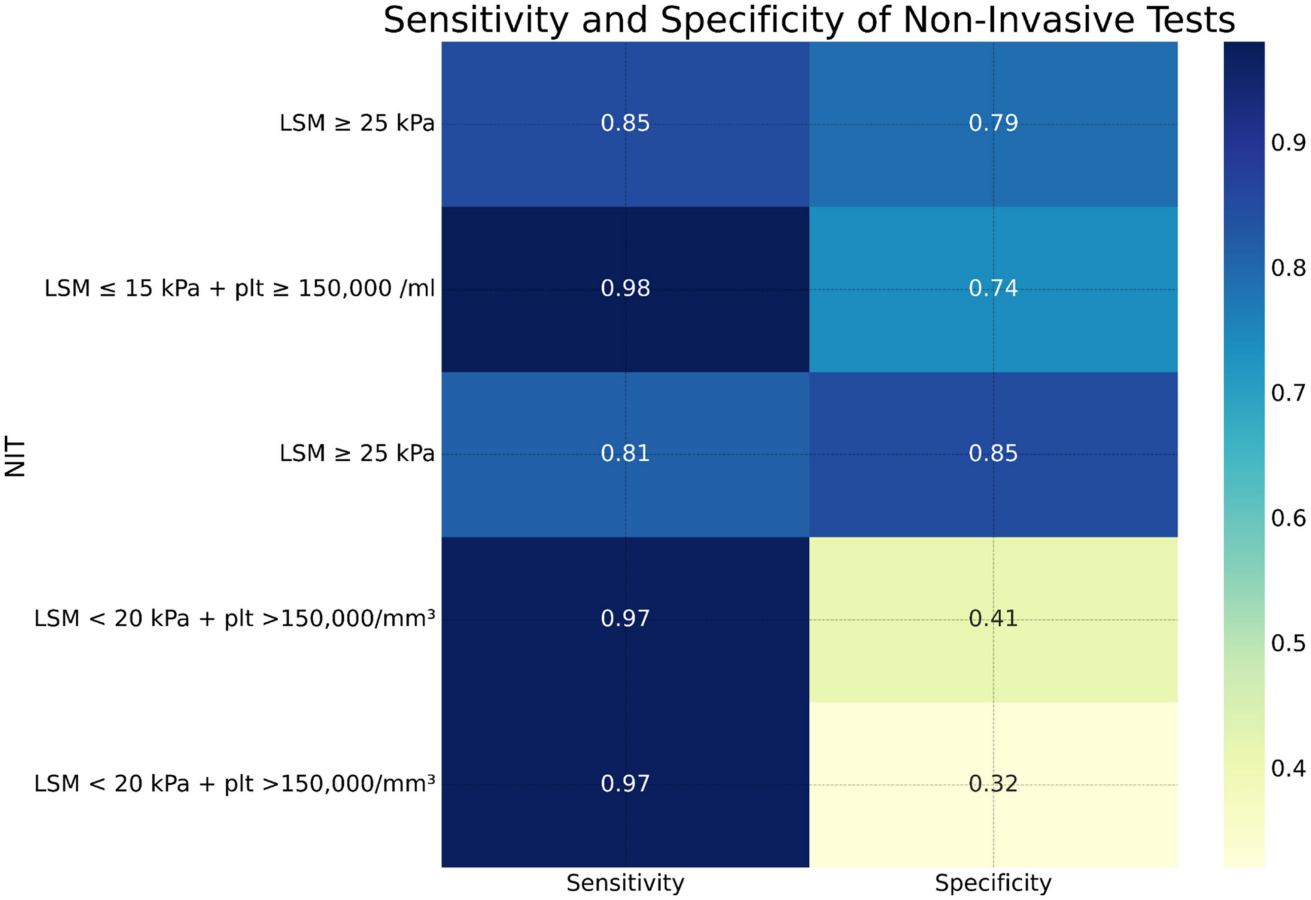
Heatmap showing sensitivity and specificity of various non-invasive tests for diagnosing clinically significant portal hypertension (CSPH), severe portal hypertension (SPH), esophageal varices (EV), and high-risk esophageal varices (HREV).

Forest plots were generated to visually represent the pooled diagnostic accuracy metrics, allowing a clear comparison of the sensitivity and specificity across the included studies. Figures 3, 4 show the forest plots for the diagnostic performance of LSM in detecting clinically significant portal hypertension (CSPH) and esophageal varices (EV), respectively (32).

In the case of CSPH, the forest plot (Figure 3) demonstrated a consistent diagnostic sensitivity across studies, with some variability in specificity. The pooled sensitivity of LSM was calculated to be 85% (95% confidence interval [CI]: 82–89%), while the pooled specificity was 79% (95% CI: 74–82%). The study by Odriozola et al. (22) contributed the highest sensitivity (97%), while Rana et al. (24) showed the highest specificity (80%) (33).

For variceal bleeding, the forest plot for LSM (Figure 4) revealed a pooled sensitivity of 88% (95% CI: 84–92%) and a pooled specificity of 70% (95% CI: 64–74%). The combination of LSM with platelet count was particularly effective in identifying high-risk esophageal varices (HREV), with the highest sensitivity observed in the Odriozola et al. (22) study, reaching 97%.

### 3.5 Heterogeneity and subgroup analysis

Significant heterogeneity was observed across the included studies, particularly in terms of patient demographics (e.g., age, sex, BMI) and study design (e.g., prospective vs. retrospective). The *I*^2^ statistic for heterogeneity was calculated to be 62% for sensitivity and 58% for specificity in the CSPH analysis, indicating moderate heterogeneity. In the case of variceal bleeding, heterogeneity was slightly higher, with an *I*^2^ value of 65% for sensitivity and 63% for specificity (34).

To explore potential sources of heterogeneity, subgroup analyses were performed. These analyses revealed that patients with more advanced fibrosis (F3–F4) exhibited slightly lower diagnostic specificity for LSM, likely due to greater hepatic stiffness variability at more severe stages of liver disease. Similarly, patients with comorbid conditions such as metabolic syndrome or higher BMI showed lower overall diagnostic accuracy of both LSM and SSM. This finding highlights the need for tailored diagnostic approaches in specific patient populations (12, 30).

### 3.6 Meta-regression and sensitivity analyses

Meta-regression analyses revealed that higher BMI and advanced liver fibrosis were associated with reduced sensitivity and specificity of LSM, suggesting that patient characteristics significantly influence test performance. Sensitivity analyses, which excluded studies with high risk of bias, confirmed the robustness of the primary findings. The exclusion of these studies did not significantly alter the pooled estimates of sensitivity and specificity, indicating the stability of the results (31).

### 3.7 Sensitivity analysis and robustness of results

Sensitivity analyses were conducted by excluding studies with a high risk of bias, as determined by the QUADAS-2 tool. The robustness of the pooled estimates was confirmed, with only minor fluctuations in diagnostic accuracy metrics after excluding these studies. For instance, the pooled sensitivity of LSM for CSPH remained consistent at 85%, while the pooled specificity showed a slight improvement, increasing from 79 to 81%.

Overall, the results of this systematic review indicate that non-invasive methods, particularly LSM and SSM, offer high diagnostic accuracy for detecting both portal hypertension and variceal bleeding in patients with NAFLD/MASLD cirrhosis. These findings are robust, with sensitivity analyses confirming the reliability of the primary outcomes.

## 4 Discussion

The present systematic review aimed to evaluate the diagnostic accuracy of non-invasive methods for diagnosing portal hypertension and variceal bleeding in patients with liver cirrhosis secondary to NAFLD, recently redefined as metabolic dysfunction-associated steatotic liver disease (MASLD). By analyzing 11 studies comprising a total of 2,707 patients, our findings provide a comprehensive perspective on the current evidence regarding non-invasive techniques, particularly liver stiffness measurement (LSM) and spleen stiffness measurement (SSM), as diagnostic alternatives to invasive methods such as hepatic venous pressure gradient (HVPG) and esophagogastroduodenoscopy (EGD). This discussion will integrate and analyze the main findings from our review, address clinical implications, compare the results with existing literature, discuss limitations, and suggest directions for future research (39).

### 4.1 Main findings

Our analysis identified LSM, particularly when combined with platelet count, as a highly sensitive diagnostic tool for identifying clinically significant portal hypertension (CSPH) and high-risk esophageal varices (HREV) in patients with NAFLD/MASLD cirrhosis. The pooled sensitivity of LSM for detecting CSPH was 85%, with a specificity of 79% at a cutoff value of 20 kPa. These findings are consistent across multiple studies, confirming the diagnostic utility of LSM as a primary screening tool. For severe portal hypertension (SPH), LSM demonstrated a sensitivity of 81% and specificity of 85% at a cutoff of 25 kPa, reflecting its value for both ruling out and confirming CSPH (22, 26).

When combined with platelet count, LSM reached a high sensitivity (97%) but showed reduced specificity (41%) for CSPH. This combination provides a strong diagnostic approach for ruling out CSPH, particularly in resource-limited settings where reducing the need for confirmatory invasive testing can reduce healthcare costs and patient discomfort. SSM also demonstrated high sensitivity (89%) and specificity (75%) for CSPH at a threshold of 40 kPa. SSM’s diagnostic performance was comparable to LSM, making it a promising tool, particularly for patients with contraindications to LSM or in centers where SSM is more accessible (27).

In terms of detecting variceal bleeding, LSM’s pooled sensitivity was 88% and specificity was 70%, with higher accuracy in detecting HREV. SSM was similarly effective in identifying high-risk varices, demonstrating a sensitivity of 85% and specificity of 78%. These results suggest that both LSM and SSM, particularly when combined with platelet count or other composite scores, offer reliable diagnostic alternatives for assessing the risk of variceal bleeding in patients with MASLD cirrhosis (28, 29).

### 4.2 Clinical implications

The findings of this review suggest that non-invasive tests, especially LSM in combination with platelet count, have high sensitivity for diagnosing CSPH and HREV in patients with NAFLD/MASLD cirrhosis. These tools have the potential to reduce the need for invasive procedures, particularly in settings with limited access to HVPG measurement and EGD. Further research should focus on validating these findings across different clinical settings and patient populations to establish standardized diagnostic protocols (29, 30).

### 4.3 Comparison with existing literature

Our results align with previous systematic reviews that have highlighted LSM as a reliable, non-invasive alternative to traditional methods for assessing portal hypertension in liver cirrhosis (25, 35). For instance, Manatsathit et al. (35) demonstrated that LSM had high diagnostic accuracy for CSPH in patients with liver cirrhosis, findings corroborated by our analysis and by subsequent studies that evaluated LSM combined with other non-invasive parameters such as platelet count (24). Recent studies also underscore the value of SSM, particularly in light of the limitations of LSM in patients with obesity or ascites, further supporting our findings on SSM’s reliability as a complementary diagnostic tool (26, 27).

However, our review also highlights certain challenges and inconsistencies in the existing literature. Studies included in our analysis reported variability in the diagnostic thresholds used for LSM and SSM, with cutoff values for CSPH ranging from 15 to 25 kPa. This lack of standardization contributes to heterogeneity in diagnostic accuracy and underscores the need for consensus guidelines on cutoff values. Additionally, while composite scores combining LSM, platelet count, and spleen diameter-to-platelet ratio have shown promise, the variability in study methodologies and patient characteristics suggests that further validation is needed to confirm the reliability of these scores across diverse patient populations (11, 29).

### 4.4 Limitations of the current review

Several limitations should be noted in interpreting the results of this systematic review. First, significant heterogeneity was observed across studies, which may reflect variations in patient demographics (such as age, sex, BMI, and liver disease severity), diagnostic test protocols, and geographic settings. While we conducted subgroup and sensitivity analyses to account for these differences, the moderate to high heterogeneity in some outcomes suggests that our findings should be interpreted with caution (30, 34).

Second, most studies included in this review were conducted in specialized centers with access to advanced diagnostic equipment and trained personnel, which may limit the generalizability of our findings to community or resource-limited settings. Furthermore, the majority of the studies were observational, with relatively few randomized controlled trials (RCTs), which restricts our ability to establish causative relationships between non-invasive test results and clinical outcomes (12, 34).

Finally, while our meta-analysis focused primarily on diagnostic accuracy, it did not extensively address the impact of non-invasive diagnostic strategies on patient outcomes, such as the rate of progression to variceal bleeding or liver-related mortality. Future research should aim to evaluate the clinical impact of non-invasive testing on these outcomes, particularly as a means of validating the role of LSM and SSM in routine clinical practice (33, 34).

### 4.5 Future research directions

Our findings underscore the need for continued research to optimize non-invasive diagnostic methods for portal hypertension and variceal bleeding in patients with MASLD cirrhosis. Future studies should focus on establishing standardized diagnostic thresholds for LSM, SSM, and other composite scores to reduce variability and enhance the reliability of these tests across diverse populations. Large-scale, multicenter RCTs evaluating the impact of non-invasive diagnostic pathways on patient outcomes are also needed to confirm the utility of these methods in clinical practice (12, 34).

Additionally, future research should explore the integration of novel biomarkers and imaging modalities with LSM and SSM to improve diagnostic accuracy. For instance, serum biomarkers such as aspartate aminotransferase-to-platelet ratio index (APRI) and fibrosis-4 index (FIB-4) have shown promise in assessing liver fibrosis and may enhance the predictive value of LSM when combined in a diagnostic algorithm (29, 30). Machine learning approaches could also be applied to non-invasive diagnostic data to develop predictive models for CSPH and variceal bleeding, enabling personalized, risk-based screening strategies.

Lastly, given the recent reclassification of NAFLD to MASLD, future studies should specifically address the implications of this new nomenclature on disease characterization and diagnostic approaches. As metabolic syndrome becomes increasingly prevalent, evaluating the impact of comorbid conditions such as diabetes, obesity, and hypertension on the accuracy of non-invasive diagnostic methods will be essential to refining diagnostic and therapeutic strategies for MASLD-related liver disease (26, 29).

## 5 Conclusion

This systematic review highlight the effectiveness of non-invasive diagnostic methods, particularly liver stiffness measurement (LSM) and spleen stiffness measurement (SSM), in diagnosing portal hypertension and variceal bleeding in patients with liver cirrhosis secondary to non-alcoholic fatty liver disease (NAFLD), now more appropriately termed metabolic dysfunction-associated steatotic liver disease (MASLD). Through a detailed analysis of 11 studies encompassing 2,707 patients, we have established that non-invasive methods demonstrate high diagnostic accuracy, especially for clinically significant portal hypertension (CSPH) and severe portal hypertension (SPH). LSM, with a sensitivity of 85% and specificity of 79% at a 20 kPa cutoff, has emerged as a valuable tool for identifying CSPH, while SSM provides comparable diagnostic performance and serves as a strong alternative or complement to LSM (24, 26).

The study also emphasizes the significant potential of combining LSM with platelet count for diagnosing CSPH, which enhances diagnostic sensitivity to 97% while maintaining reasonable specificity. Such a combination could be crucial for use in clinical settings where reducing the need for invasive tests like hepatic venous pressure gradient (HVPG) measurement or esophagogastroduodenoscopy (EGD) is desirable due to resource limitations, patient preference, or clinical constraints. In terms of identifying variceal bleeding, both LSM and SSM provide substantial diagnostic accuracy, particularly for high-risk esophageal varices (HREV), suggesting that these methods could play an essential role in determining which patients would benefit most from surveillance and intervention (28, 29).

However, our findings underscore the need for standardizing diagnostic protocols across clinical settings. The variability in cutoff values across studies for both LSM and SSM highlights a gap that needs to be addressed through consensus guidelines and further research. Additionally, significant heterogeneity in patient demographics, study design, and geographical factors suggests that individual patient factors, such as metabolic syndrome, obesity, and liver disease severity, may influence the diagnostic accuracy of these non-invasive tools. Consequently, while LSM and SSM demonstrate high potential for integration into clinical practice, further validation and refinement of these methods are necessary to ensure consistent and accurate diagnosis across diverse populations (31, 34).

In summary, non-invasive tests like LSM and SSM represent a transformative step toward reducing reliance on invasive procedures for diagnosing complications in MASLD-related cirrhosis. By providing accurate, accessible, and patient-friendly alternatives, these methods have the potential to enhance early detection, optimize patient management, and reduce healthcare costs associated with invasive diagnostics. The findings of this review strongly support their integration into clinical pathways, particularly for screening and risk stratification in MASLD cirrhosis (28, 29).

## 6 Recommendations

### 6.1 Clinical practice recommendations

Integration of non-invasive tests into routine clinical practice: LSM and SSM should be incorporated as first-line screening tools for diagnosing CSPH and assessing the risk of variceal bleeding in patients with MASLD cirrhosis. For instance, patients with LSM values below 20 kPa and platelet counts above 150,000/mm^3^ can be considered low risk, allowing clinicians to potentially avoid invasive diagnostic procedures (26, 29).

Personalized diagnostic approaches: Considering the influence of factors such as body mass index (BMI), metabolic syndrome, and comorbidities on the accuracy of LSM and SSM, clinicians should adopt a personalized approach. This could involve selecting appropriate cutoff values or combining non-invasive tests with other clinical markers to improve diagnostic accuracy in individual patients (29, 30).

Use of composite scores for enhanced accuracy: For patients who may benefit from additional diagnostic precision, combinations of LSM, platelet count, and SSM can be used to increase diagnostic accuracy. Composite scores like the liver stiffness-spleen diameter to platelet ratio score (LSPS) offer an approach for identifying patients with high-risk esophageal varices who may require closer monitoring or prophylactic intervention (28, 29).

Regular monitoring and follow-up: Non-invasive tests such as LSM and SSM can serve as part of a monitoring regimen for patients with MASLD cirrhosis, allowing for timely detection of disease progression. Regular monitoring can guide changes in patient management, such as escalating therapy or preparing for interventional procedures if non-invasive parameters indicate increasing risk of complications (31).

Improving patient education and compliance: Given the non-invasive nature of LSM and SSM, these tests provide an opportunity to engage patients in regular monitoring with minimal discomfort or risk. Educating patients on the value and reliability of these tests may enhance adherence to follow-up protocols and improve long-term outcomes by facilitating timely intervention (34).

### 6.2 Recommendations for future research

Standardization of diagnostic thresholds: To address the variability in cutoff values across studies, future research should aim to establish standardized thresholds for LSM and SSM in diagnosing CSPH and SPH in MASLD cirrhosis. Large-scale multicenter studies across diverse patient populations will be essential to developing universally applicable guidelines (30).

Prospective studies and randomized controlled trials (RCTs): There is a need for prospective studies and RCTs evaluating the impact of non-invasive diagnostic strategies on clinical outcomes, such as progression to variceal bleeding, liver-related mortality, and quality of life. These studies should assess not only diagnostic accuracy but also the potential benefits of non-invasive tests in reducing complications and healthcare costs (12, 29).

Development of novel biomarkers and composite scores: Integrating new biomarkers, such as the aspartate aminotransferase-to-platelet ratio index (APRI) or fibrosis-4 index (FIB-4), with existing non-invasive methods could further improve diagnostic accuracy. Additionally, composite scores that combine multiple non-invasive parameters, machine learning models, and clinical risk factors may provide highly individualized diagnostic insights (29, 31).

Longitudinal studies on disease progression and intervention needs: Long-term follow-up studies are needed to evaluate the effectiveness of non-invasive tests in predicting disease progression and guiding intervention timing. Such studies would be valuable in understanding how non-invasive diagnostic approaches can be optimized to prevent the progression of MASLD cirrhosis and reduce the incidence of variceal bleeding and other complications (28, 29).

Cost-effectiveness analysis: Research is also needed to determine the economic impact of integrating non-invasive diagnostic methods into clinical pathways for MASLD cirrhosis. Comparative studies on the costs associated with invasive versus non-invasive approaches could support broader adoption of LSM, SSM, and composite scores as primary diagnostic tools, especially in settings where healthcare resources are limited (12, 34).

Evaluation in diverse patient populations: Future research should focus on validating the findings in diverse populations with varying metabolic profiles and risk factors, including patients with metabolic syndrome, diabetes, and obesity. Such studies would ensure that non-invasive diagnostic methods are applicable to all MASLD patients, regardless of comorbidities or regional differences in disease presentation (26, 29).

In conclusion, while non-invasive methods for diagnosing portal hypertension and variceal bleeding in MASLD-related cirrhosis show substantial promise, further research is essential to maximize their diagnostic accuracy, establish universal guidelines, and evaluate their impact on patient outcomes. Standardizing these methods and incorporating them into clinical practice could significantly improve patient care, reduce healthcare costs, and enhance early intervention strategies for MASLD cirrhosis on a global scale (14, 37).

## Data Availability

The original contributions presented in the study are included in the article/supplementary material, further inquiries can be directed to the corresponding author/s.

## Footnotes

^1^ http://www.crd.york.ac.uk/PROSPERO
